# MicroRNA-146b-3p Regulates Retinal Inflammation by Suppressing Adenosine Deaminase-2 in Diabetes

**DOI:** 10.1155/2015/846501

**Published:** 2015-03-01

**Authors:** Sadanand Fulzele, Ahmed El-Sherbini, Saif Ahmad, Rajnikumar Sangani, Suraporn Matragoon, Azza El-Remessy, Reshmitha Radhakrishnan, Gregory I. Liou

**Affiliations:** ^1^Department of Orthopedics, Georgia Regents University, Augusta, GA, USA; ^2^Department of Ophthalmology, Georgia Regents University, Augusta, GA 30912, USA; ^3^Rabigh College of Science and Arts, King Abdulaziz University, Jeddah, Saudi Arabia; ^4^Program in Clinical and Experimental Therapeutics, University of Georgia, Augusta, GA, USA

## Abstract

Hyperglycemia- (HG-) Amadori-glycated albumin- (AGA-) induced activation of microglia and monocytes and their adherence to retinal vascular endothelial cells contribute to retinal inflammation leading to diabetic retinopathy (DR). There is a great need for early detection of DR before demonstrable tissue damages become irreversible. Extracellular adenosine, required for endogenous anti-inflammation, is regulated by the interplay of equilibrative nucleoside transporter with adenosine deaminase (ADA) and adenosine kinase. ADA, including ADA1 and ADA2, exists in all organisms. However, because ADA2 gene has not been identified in mouse genome, how diabetes alters adenosine-dependent anti-inflammation remains unclear. Studies of pig retinal microglia and human macrophages revealed a causal role of ADA2 in inflammation. Database search suggested miR-146b-3p recognition sites in the 3′-UTR of ADA2 mRNA. Coexpression of miR-146b-3p, but not miR-146-5p or nontargeting miRNA, with 3′-UTR of the ADA2 gene was necessary to suppress a linked reporter gene. In the vitreous of diabetic patients, decreased miR-146b-3p is associated with increased ADA2 activity. Ectopic expression of miR-146b-3p suppressed ADA2 expression, activity, and TNF-*α* release in the AGA-treated human macrophages. These results suggest a regulatory role of miR-146b-3p in diabetes related retinal inflammation by suppressing ADA2.

## 1. Introduction

Diabetic retinopathy (DR) is a leading cause of blindness among working-age adults. Treatment options for DR remain limited and with adverse effects. Major complications in DR include blood-retinal barrier dysfunction and loss of retinal neurons [1–3]. Although these changes may be a major vision-threatening complication in diabetes, by the time they become easily demonstrable, tissue damage has already occurred. Therefore, there is a great need for early detection and intervention of DR during the prediabetic phase.

During early diabetes, retinal immune cell activation causes retinal inflammation leading to major DR complications. These cells are involved in proinflammatory as well as anti-inflammatory processes. Anti-inflammatory process may be induced by extracellular adenosine that activates adenosine receptors (A1AR, A2AAR, A2BAR, and A3AR). A2AAR, a Gs-coupled adenosine receptor, plays a major role in anti-inflammation. Extracellular concentrations of adenosine are regulated by the interplay of the equilibrative nucleoside transporter (ENT) with intra- and extracellular enzymes of adenosine metabolism. Extracellular adenosine and 2′-deoxyadenosine can be internalized through ENT and deaminated to inosine and deoxyinosine by ADA. Two different isoenzymes of ADA, designated as ADA1 and ADA2, were found in mammals, lower vertebrates, and insects [4]. ADA1 is ubiquitous and is critical for the downregulation of adenosine and 2′-deoxyadenosine [5]. Unlike ADA1, the extracellular ADA2 shows a weak affinity for 2′-deoxyadenosine. During inflammation, an increase in ADA2 has been found in macrophage-rich tissues [6, 7]. ADA2 activity is elevated significantly in pleural fluids of patients with pulmonary tuberculosis [8], sera from HIV-infected individuals [9, 10], and from patients with diabetes [11], making ADA2 activity a convenient marker to improve the diagnosis and follow-up treatment of these disorders. In contrast to ADA1, ADA2 activity for adenosine requires high levels of adenosine and low optimum pH of 6.5, suggesting that ADA2 expresses its activity only at conditions that are associated with hypoxia or inflammation [4]. It was shown that ADA2 is important for monocyte differentiation and stimulation of macrophage proliferation [12]. The search for a rodent ADA2 gene by analysis at the critical region (at or near the human chromosome 22 pericentromere) in humans and the region of conserved synteny in mice has not been successful [13, 14]. The role of ADA2, therefore, has been understudied in mice as the sequencing probes or antibodies to mouse ADA2 are not available [15]. To determine the role of ADA2 in diabetes, the treatment effects of Amadori-glycated albumin (AGA) [2] or HG on the porcine retinal microglia and human monocytes/macrophages (U937) were determined. In the AGA-treated cells, increased ADA2 expression, ADA2 activity, and TNF-*α* release were induced and these effects were blocked by ADA2-neutralizing antibody or ADA2 siRNA but not by scrambled siRNA [16]. These results suggest that retinal inflammation in DR is mediated by ADA2 and that the anti-inflammatory activity of adenosine receptor signaling is impaired in diabetes due to increased ADA2 activity.

A number of factors regulate gene expression at the transcriptional and translational levels during developmental and diseased conditions. MicroRNAs (miRNAs) are a class of small noncoding RNA molecules, 22 to 25 nucleotides in length that function in the posttranscriptional regulation of gene expression. miRNAs bind partly complementary sequences in mRNAs, targeting them for degradation and/or inhibiting their translation and thereby downregulating the expression of the targeted proteins [17–19]. Dysregulation of miRNAs has been shown to contribute to many types of human diseases, including neuronal disorders [15, 18, 19]. miR-146b-5p, an miRNA regulated by variable globular adiponectin concentrations and acting as an inhibitor of NF*κ*B-mediated inflammation, is decreased in circulating monocytes of obese subjects with type 2 diabetes [15, 20]. miR-146b-5p and miR-146b-3p are two isoforms from the same gene in human chromosome 10 with different sequences and for the regulation of different gene expression. In contrast to the well-studied miR-146b-5p, the targets of miR-146b-3p involved in diabetes are not well studied. In the current study, we demonstrate that ADA2 is one of the targets of miR-146-3p. We identified a role of miR-146b-3p in the regulation of retinal inflammation in diabetes by suppressing ADA2.

## 2. Materials and Methods

### 2.1. Postmortem Eye Specimens

Sixteen human eyes, including 8 globes of type 2 diabetes and 8 nondiabetic controls, were obtained from Georgia Eye Bank (Atlanta, GA) according to the following selection criteria: >50 years old, either insulin requiring diabetes or no diabetes, and no life-support measures. The eyes were enucleated an average of 6.71 ± 0.84 h after death. Similarly collected postmortem eye specimens were used in our previous studies [21].

### 2.2. Immunofluorescence Labeling of Paraffin-Embedded Human Eyes

A microwave oven-based technique for immunofluorescent staining of paraffin-embedded tissues [22] was used to doubly label human eye sections with antibodies for Iba1 (WAKO, code number 016-20001) and ADA2 (Santa Cruz, catalogue number sc-86100).

### 2.3. ADA2 Activity Assay

The ADA2 assay is based on the enzymatic deamination of adenosine at pH 6 to inosine, which is converted to hypoxanthine by purine nucleoside phosphorylase. Hypoxanthine is then converted to uric acid and hydrogen peroxide (H_2_O_2_) by xanthine oxidase. H_2_O_2_ is further reacted with N-ethyl-*N*-(2-hydroxy-3-sulfopropyl)-3-methylaniline and 4aminoantipyrine in the presence of peroxidase to generate quinone dye, which is monitored in a kinetic manner. ADA1 activity is inhibited by ADA1-specific inhibitor erythro-9-(2-hydroxy-3-nonyl) adenine (EHNA) (Diazyme Laboratories, Poway, CA).

### 2.4. Drug Treatment Effects on the Cultured Human Macrophages

The human monocyte cell line U937 was grown in ATCC formulated RPMI-1640 medium (catalog number 30-2001) containing 10% fetal bovine serum (Atlanta Biologicals), subcultured in GIBCO RPMI-1640 (Catalog number 11879-020, supplemented with 5 mM D-glucose), and differentiated into macrophages by phorbol 1 2-myristate 13-acetate (PMA, 50 ng/mL) treatment. Cells were then cultured in the same media supplemented with AGA (Sigma, St Louis, MO) which contained undetectable endotoxin (<0.125 units/mL, 10 EU = 1 ng lipopolysaccharide; Lonza, Basel, Switzerland) at a final concentration of 500 mg/mL for 12 hours [2]. Cells were also cultured in HG (GIBCO RPMI-1640 supplemented with 35 mM D-glucose) or in osmotic pressure-control medium (GIBCO RPMI-1640 supplemented with 10 mM D-glucose and 25 mM L-glucose) for 48 hours.

### 2.5. Human Monocytes Transfection

The human monocyte cell line U937 was cultured in ATCC formulated RPMI-1640 medium (catalog number 30-2001), differentiated into macrophages by PMA treatment at 50 ng/mL. Nonviral transfection of cells was performed using LonzaH NucleofectorH II electroporation system. An aliquot of 10^6^ U937 cells were pelleted and resuspended in 100 *μ*L electroporation buffer (VCA-1004) containing negative control (NC), miR-146-5p mimic, or miR-146b-3p mimic at a final concentration of 100 nM. The cells were immediately transferred to a cuvette and electroporated using the program W-001. After transfection, cells were resuspended in medium and grown overnight. The human monocyte cell line was also transfected by lipofectin using Lipofectamine 2000 (Invitrogen) as described by the manufacturer [23].

### 2.6. Vector Construction and Luciferase Reporter Assay

For luciferase reporter analyses, the 3′-UTR of the human ADA2 gene (2184 bp) amplified by PCR from human cDNA was cloned into the pEZX-MT05 (GeneCopoeia, Rockville, MD). U937 cells were seeded into 12-well plates (5 × 10^5^ cells/well), PMA-differentiated, and transfected using nucleofector technology described above. Cells were cotransfected with 100 nM miR-146b-3p mimic, miR-146b-5p mimic, or NC and 1 *μ*g of pEZX-MT05 with or without the 3′-UTR of the human ADA2 gene. After 24 h, culture media were refreshed and after another 48 h, activities of Gaussia luciferase (GLuc) and secreted alkaline phosphatase (SEAP) were determined with a luminometer. The relative reporter activity was obtained by normalizing the GLuc activity against SEAP activity.

### 2.7. Quantitative Real-Time PCR for mRNA and miRNA

For mRNA quantitation, SV Total RNA Isolation kit (Promega) was used for total RNA isolation. RNA was reverse-transcribed into cDNA using iScript reagents (Bio-Rad). Fifty ng of cDNA was amplified in each qRT-PCR using SYBR Green I and appropriate primers. Average of glyceraldehyde-3-phosphate dehydrogenase and 18S rRNA were used as the internal control for normalization. For miR-146b-3p and miR-146b-5p quantitation, total RNA isolated from cells or vitreous were reverse-transcribed into cDNA using miScript reagents (Qiagen). Fifty pg of cDNA was amplified in each qRT-PCR using SYBR Green I and miR-146b-3p or miR-146b-5p primer. HsRNU6 was used as normalization reference gene for miRNA.

### 2.8. ELISA for TNF-*α*

TNF-*α* levels in the supernatants of culture media were estimated with ELISA kits (R & D, Minneapolis, MN) per the manufacturer's instructions. Standards and samples were added and bound by the immobilized antibody. After washing an enzyme-linked polyclonal antibody specific for the cytokine was added to the wells followed by a substrate solution yielding a colored product. The intensity of the color was measured at 450 nm. The sample levels were calculated from the standard curve and corrected for protein concentration.

### 2.9. Statistical Analysis

The results are expressed as mean ± SD. Differences among experimental groups were evaluated by analysis of variance, and the significance of differences between groups was assessed by the post hoc test (Fisher's PLSD). Significance was defined as *P* < 0.05.

## 3. Results and Discussion

### 3.1. Localization of ADA2 in the Human Retina with Diabetes

**Figure 1:**
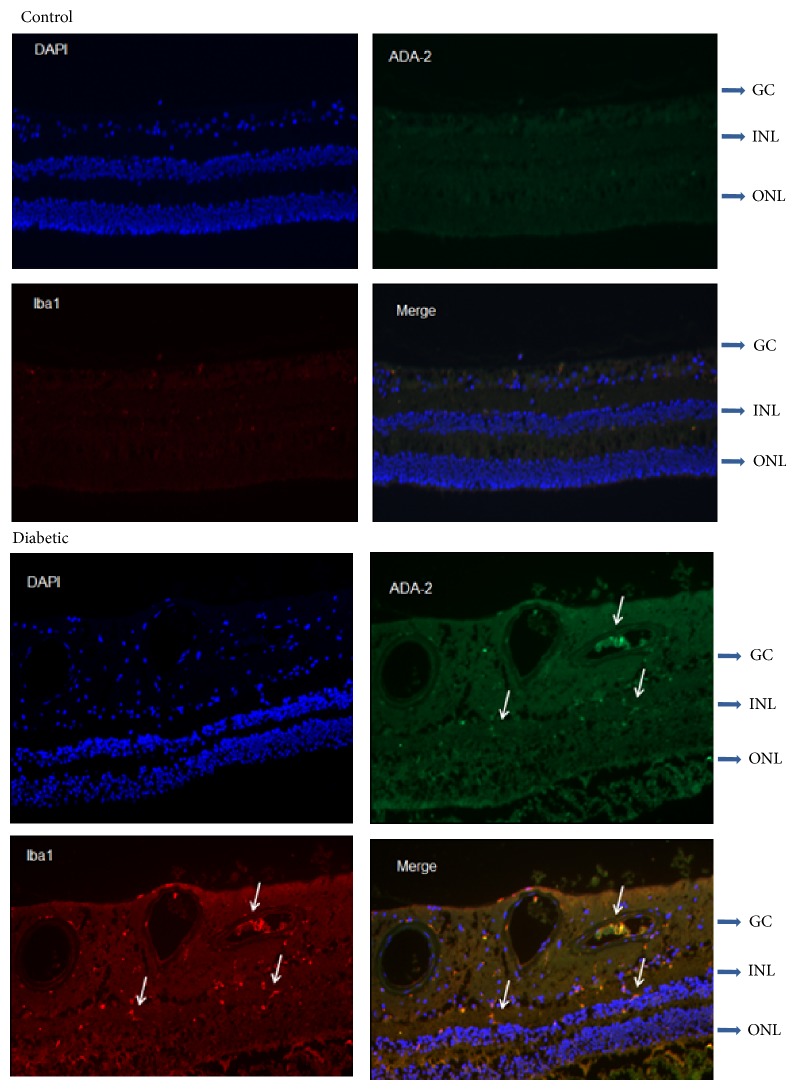
ADA2 is localized in the activated monocytes or macrophages in human retina with diabetes. Immunolabeling of ADA2 (green) and Iba-1 (red), a marker of activated microglia or macrophages, was made in human retinas under normal and diabetic conditions. Colocalization of ADA2 and Iba1, as marked by the orange-stained cells in the inner-retina produced by the merged red and green on the cell surface, is only identified in retinas with diabetes. The figures represent one of three donor eyes in each diabetic and normal group.

During inflammation, increased ADA2 has been found in macrophage-rich tissues [6, 7]. This suggests that ADA2 is involved in inflammation in the macrophages. To investigate the role of ADA2 in the retina during diabetes, we sought to determine cellular localization of ADA2 in the human retina with and without diabetes (*n* = 3 each). Donor eyes with or without type 2 diabetes were paraffin-embedded and sectioned. Immunolabeling was processed for ADA2- and Iba-1-positive cells using antibodies specific for human ADA2 and Iba-1, respectively. Colocalization of ADA2 and Iba1, as indicated by the merged yellow fluorescence on cell surface (*arrows*), is identified in retinas with diabetes but not nondiabetic control (Figure 1). This result suggests that ADA2 upregulation occurs in the activated microglia or macrophages in the diabetic retina.

### 3.2. ADA2 Is a Direct Target of miR-146b-3p

**Figure 2:**
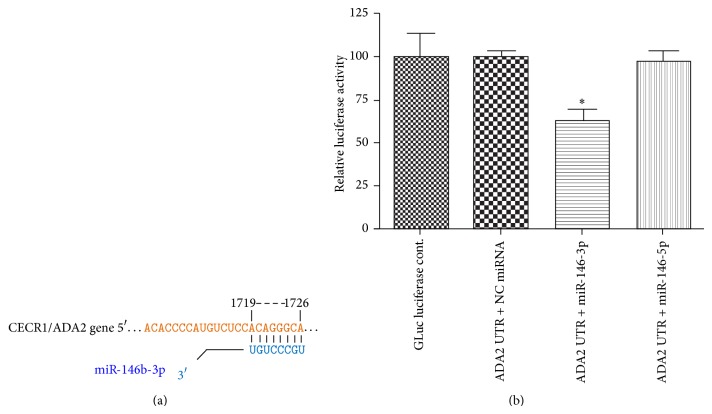
ADA2 is a target of miR-146b-3p. (a) Database searches of the TargetScan sites showed that human ADA2 mRNAs have conserved miR-146b-3p recognition sites. Alignment of the predicted miRNA binding sites in the 3′-UTR of the human ADA2 mRNA was made. Pairing of target region (top) and miRNA (bottom) is shown. (b) Secrete-Pair Dual Luminescence assay system was used to determine if ADA2 expression might be suppressed directly by miR-146b-3p. GLuc luciferase reporter gene linked with or without the 3′-UTR of the ADA2 gene was cotransfected with miR-146b-3p mimic, miR-146b-5p mimic, or negative control (NC, nontargeting miRNA) in differentiated U937 cells. Luciferase activities normalized with SEAP activities were determined with a luminometer. The results are mean ± SD from three independent experiments. ^*^ *P* < 0.05.

miRNAs play a major role as negative regulators of protein-coding genes. To determine the role of ADA2 in the retina under the condition of diabetes, we sought to identify miRNA that targets ADA2. TargetScan predicts biological targets of miRNAs by searching for the presence of conserved 8mer and 7mer sites that match the seed region of each miRNA [24]. Database searches of the TargetScan sites showed that human ADA2 mRNAs have conserved miR-146b-3p recognition sites (Figure 2(a)). We hypothesize that increased ADA2 production might result from the downregulation of miR-146b-3p. To test this hypothesis, a Secrete-Pair Dual Luminescence assay system (GeneCopoeia) was used in U937 cells. Coexpression of miR-146b-3p mimic, but neither miR-146b-5p mimic nor negative control (NC), significantly suppressed the GLuc luciferase reporter activity of the linked 3′-UTR, indicating that miR-146b-3p suppresses ADA2 expression through miRNA binding [20] sequences in its 3′-UTR (Figure 2(b)). Together, these results suggest that miR-146b-3p suppresses ADA2 expression by binding to the 3′-UTR and that ADA2 is a direct target of miR-146b-3p.

### 3.3. miR-146b-3p Is Dysregulated in Diabetes

**Figure 3:**
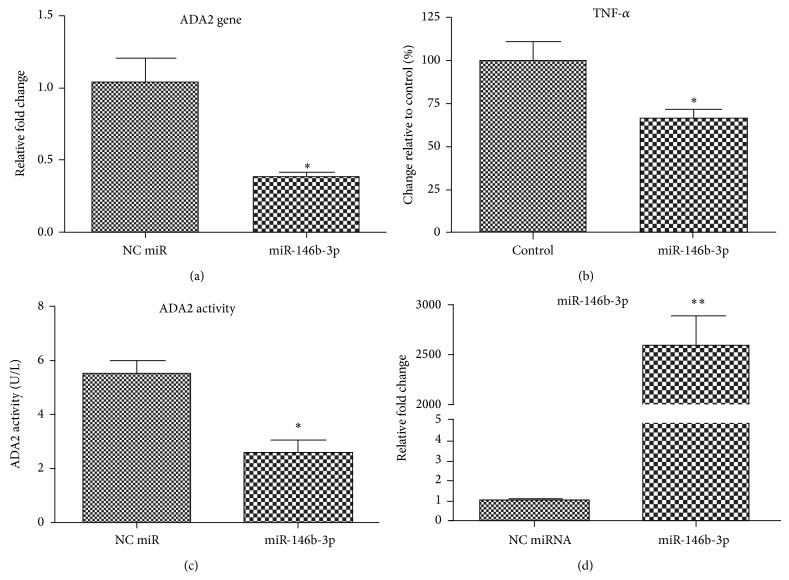
miR-146b-3p is dysregulated in diabetes: ectopic expression of miR-146b-3p inhibits ADA2 and TNF-*α* expression. PMA-differentiated U937 macrophages were transfected with miR-146b-3p mimic or negative control (NC, nontargeting miRNA). Expression was evaluated by qRT-PCR for (a) ADA2 mRNA and (b) TNF-*α* quantification (ELISA). (c) ADA2 activity using standard quantification method and (d) miR-146b-3p expression (qRT-PCR). The results are presented as the mean ± SD from three independent experiments. ^*^ *P* < 0.05.

Dysregulation of miRNAs has been shown to contribute to many types of human diseases, including diabetes [15] and neuronal disorders [18, 19]. To determine whether miR-146-3p is dysregulated in diabetes, we first determined the effect of miR-146b-3p overexpression in PMA-differentiated U937 monocytes. PMA-treated cells were transfected with nucleofector technology with negative control (NC), miR-146b-3p mimic, and exposed to AGA. ADA2 expression, activity, and TNF-*α* release were determined by qRT-PCR, standard assay, and by ELISA, respectively. Evaluation of the cell lysates showed that the miR-146b-3p-transfected cells exhibited reduced ADA2 expression, activity, and TNF-*α* release (Figure 3).

### 3.4. miR-146b-3p Is Inversely Associated with ADA2 in Diabetes

**Figure 4:**
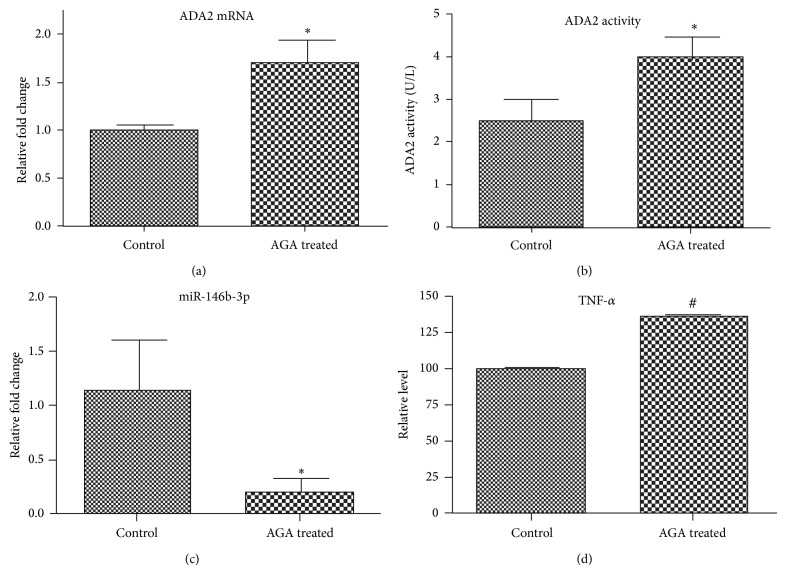
ADA2 is inversely associated with miR-146b-3p: AGA treatment upregulates TNF-*α* and ADA2 and downregulates miR-146b-3p. PMA-differentiated U937 macrophages were exposed to AGA and media and cell lysates were processed for the following analyses. (a) ADA2 activity was determined by a standard assay (Diazyme Laboratories). (b) ADA2 mRNA expression by qRT-PCR, (c) miR-146b-3p expression by qRT-PCR, and (d) TNF-*α* release were determined by ELISA. The results are mean ± SD from three independent experiments. ^*^ *P* < 0.05.

We hypothesize that as a negative regulator of ADA2, miR-146b-3p expression may be inversely associated with ADA2 expression or activity and the status of inflammation. To test this hypothesis, we measured the miR-146b-3p level, ADA2 expression, and tumor necrosis factor (TNF)-*α* release after AGA treatment of PMA-differentiated U937 monocytes. AGA, formed as glycated albumin under hyperglycemic conditions and accumulated in the retina in early diabetes, is used to mimic hyperglycemia or diabetic conditions [2]. Proinflammatory cytokines including TNF-*α*, interleukin- (IL-) 1*β*, and IL-6 have been implicated in retinal inflammation under diabetic conditions. We have recently provided evidence that TNF-*α* plays a central role in the pathogenesis of DR [25]. Therefore, TNF-*α* release was determined to represent the status of inflammation. RNA was extracted from the treated cells, and expression levels of ADA2 and miR-146b-3p were determined by quantitative (q) RT-PCR. ADA2 expression and TNF-*α* release were both significantly increased after AGA treatment (Figure 4), whereas expression of miR-146b-3p was significantly decreased after AGA treatment (Figure 4).

**Figure 5:**
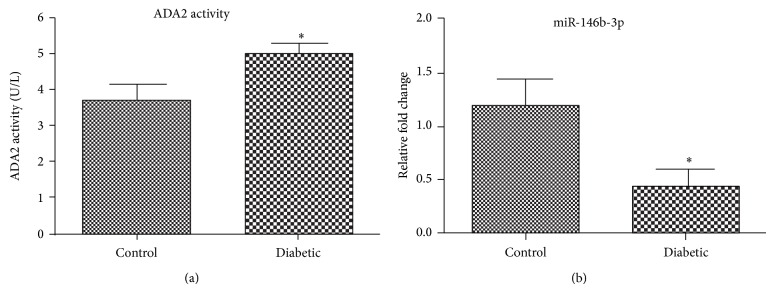
miR-146b-3p is dysregulated in diabetes: 8 donor eyes with type 2 diabetes and 4 nondiabetic donor eyes were evaluated for ADA2 activity and miR-146b-3p expression in the vitreous.^*^ *P* < 0.05.

We then determined ADA2 activity and miR-146b-3p expression in the vitreous of human donor eyes with (*n* = 8) and without diabetes (*n* = 4). The result shows that increased ADA2 activities were present in the vitreous of human donor eyes with diabetes (Figure 5). The result also shows that miR-146b-3p expression levels were downregulated in the diabetic samples after normalizing with housekeeping HsRNU6. Taken together, these results suggest that an inverse correlation between miR-146b-3p and inflammation occurs in diabetes and that reduction or dysregulation of miR-146-3p may contribute to diabetic complications.

As shown in the current study, increased ADA2 activity is accompanied with activation of macrophages and TNF-*α* release that promote retinal inflammation in diabetes. The positive regulation of ADA2 activity during inflammation is poorly understood. A number of factors such as microRNAs play important roles in regulation of genes. Our bioinformatics analysis showed that ADA2 mRNA has 3′-UTR site which is complimentary to miR-146b-3p. Our results confirm the hypothesis that miR-146b-3p binds to ADA2 3′-UTR and inhibit its expression and activity. Moreover, ectopic expression of miR-146b-3p decreases the ADA2 activity and TNF-*α* release in PMA-differentiated U937 monocytes. To verify this finding, we constructed luciferases gene containing 3′-UTR of human ADA2 gene. Luciferase reporter analysis shows that luciferase activity decreases with cotransfection of miR-146b-3p but not NC or miR-146b-5p. This study is the first that determined how ADA2 is upregulated in diabetes. The increased ADA2 activity is also associated with decreased expression of miR-146b-3p in diabetes. Dysregulation of miRNA has been shown to contribute to many types of human diseases, including neuronal disorders [18, 19]. In addition, miR-146b-5p, an miRNA regulated by variable globular adiponectin concentrations and acting as an inhibitor of NF*κ*B-, but not ADA2-mediated inflammation, is decreased in circulating monocytes of obese subjects with type 2 diabetes [15]. In contrast to miR-146b-5p, genes that are negatively regulated by miR-146b-3p in diabetes have not been identified. It is likely that, in addition to ADA2, there are other genes that are upregulated by miR-146b-3p silencing in diabetes.

Controlling endogenous adenosine signaling may represent an advantageous way in treating DR. Extracellular concentrations of adenosine are regulated by the interplay of ENT with intra- and extracellular enzymes of adenosine metabolism. We have provided experimental evidence that targeting adenosine kinase can inhibit diabetes-induced retinal abnormalities in the development of DR by potentially amplifying the endogenous therapeutic effects of site- and event-specific accumulation of extracellular adenosine [16, 26]. We have demonstrated that ADA2 has a causal role in microglial activation and retinal inflammation in diabetes. Because increased adenosine production and ADA2 activity occur at specific pathophysiological sites [6], inhibition of ADA2 activity may also represent a mechanism to selectively enhance the actions of adenosine at specific tissue sites. Inhibition of ADA2 is, therefore, potentially with limited side-effects and is of highly translational impact. Delivering an miRNA that reduces the protein levels of target genes linked to a particular disease represents a new therapeutic option. A recent study has shown that the in vivo administration of miR-124 suppresses experimental autoimmune encephalitis by affecting macrophages, suggesting that miRNA delivery could be used to treat some inflammatory diseases associated with microglial activation [19].

## 4. Conclusions

These results suggest a role of miR-146b-3p in the regulation of retinal inflammation in diabetes by suppressing ADA2. miR-146b-3p may serve as a therapeutic target for early detection and intervention of DR.

## Abbreviations

ADA2: Adenosine deaminase-2
AGA: Amadori-glycated albumin
HG: Hyperglycemia
DR: Diabetic retinopathy
ENT: Equilibrative nucleoside transporter.
